# Supplementary material for: Are carbon emissions trading and green financial instruments synergistic? -Comprehensive quantitative research based on content analysis
Source: PLoS One. 2024 Mar 7;19(3):e0298601. doi: 10.1371/journal.pone.0298601 (PMC10919601; doi:10.1371/journal.pone.0298601)
Supplement: S1 Data — (ZIP) [file pone.0298601.s001.zip › Data/地级市Z_F工作报告.zip!/║╙─╧/║╫▒┌╩╨/└┤╫╘╣1⁄2╓┌║┼í╛┬φ┐╦╩2╛▌═°í┐_2005.docx]

各位代表：

　　现在，我代表市人民政府向大会报告工作，请予审议，并请各位政协委员提出意见。

　　一、2004年工作回顾

　　一年来，市政府在省委、省政府和市委的正确领导下，团结带领全市人民，按照“思想再解放、思路再创新、效率再提高、发展再加速”的总体要求，真抓实干，奋力争先，圆满完成了市八届人大一次会议确定的各项任务。

　　国民经济进入全新发展时期。初步核算全市生产总值突破150亿元，较上年增长15.3%，是9年来最高增速；人均生产总值突破1万元，有望超过全国平均水平。财政一般预算收入5.7亿元，增长30.5%，是11年来最高增速，其中市本级3.3亿元，增长36.1%，是历史最高增速。全社会固定资产投资达到49.1亿元，增长46.8%，其中城镇投资突破40亿元，达到42.8亿元，增长57.4%，均为9年来最高增速。社会消费品零售总额34.4亿元，增长15.9%，是8年来最高增速。规模以上工业企业增加值增长22.3%，实现利润增长39.9%，综合经济效益指数提高13.3个百分点，均为7年来最高水平。金融机构各项存、贷款余额分别比年初增加13.7亿元和8.9亿元。畜牧业产值33亿元，增长19%；加工产值31亿元，增长21%，超额完成了“双30亿”的目标。粮食总产89万吨，增长16.7%，夏粮、秋粮单产均创历史新高。接待游客突破300万人次，实现旅游直接收入突破3000万元，分别增长15.2%和46.2%。

　　招商引资和大项目建设取得显著成绩。以前所未有的决心和力度推进招商引资工作。引进市外直接投资突破50亿元，较上年增长3.1倍；实际利用外商直接投资1406万美元，增长32%。落地项目687个，总投资规模152亿元，其中规模5000万元以上的38个，总投资103亿元；签约项目48个，总投资46亿元，其中5000万元以上的16个，总投资40亿元。落地项目中，73%的项目为工业项目，87%的投资为工业投资。天淇汽车模具、仙达制药、美尔斯医药中间体等项目相继投产。佳多科工贸杀虫灯、诺亚锂电池等一批具有较高技术含量的大项目落户新区。招商引资信息、项目、办法越来越多，招商引资策划能力越来越强，外来客商层次和项目质量越来越高，企业作为招商引资主体的意识和作用越来越明显。招商引资工作的快速推进，不仅大大增强了我市经济发展的活力和后劲，而且促进了干部工作作风的转变，打造了一支具有开放意识和实干精神的干部队伍。市直部门争取上级资金8.6亿元，完成年目标的141%。认真贯彻国家宏观调控政策，千方百计克服资金、用地等方面的困难，抓住机遇开工建设了一批对经济发展带动力强、符合国家产业政策的大项目。2004年底，全市超亿元在建项目20个，投资规模达到117亿元。电厂二期工程累计完成投资18亿元，三期已完成投资4.8亿元。水泥厂连续扩建二期工程顺利实现达标生产，三期完成总投资的56%。综合利用热电项目完成投资1.8亿元。采煤沉陷治理工程已下达资金计划1.75亿元。盘石头水库累计完成投资8.86亿元，大坝主体填筑完工。交通基础设施建设完成投资3.67亿元，新老区快速通道完成总投资的26%，乡镇、行政村客车通车率分别达到100%和95%。

　　各项改革取得重要进展。成功吸引了一批有实力的战略投资者参与国有企业改革，引入资金4.2亿元。对淇县龙马(集团)公司、市通用机械厂、市陶瓷厂等重点难点企业逐一攻关。筹措资金1000多万元用于解决改制困难企业敏感性债务缺口。135户企业完成改制。通过改制和吸引外资，企业活力明显增强，经营业绩明显改善，就业增加，职工工资得到提高。鹤煤(集团)公司20所学校、国营浚县农场5所学校和铁路小学移交工作已经完成。粮食流通体制改革通过积极开拓就业岗位等多项措施，平稳完成2000多名职工分流安置任务。农村信用社改革顺利推进，增扩股金1.65亿元。市、县区政府机构改革按期完成，事业单位聘用制改革基本完成，事业单位调整整合工作取得积极进展。乡镇财政管理方式改革试点工作在浚县全面铺开。在全省率先出台了政府债务资金管理暂行办法，偿还市本级债务2225万元、开发区债务6579万元。非公有制经济增加值增长18%，占全市生产总值的比重达到46.6%，较上年提高2.6个百分点。一批经营状况好、发展潜力大的民营企业脱颖而出，规模以上非公有制工业企业增加值增长40.8%。

　　城市建设快速健康推进。实现了全市城市规划集中统一管理，投入500多万元编制城市总体设计和专项规划20多项，为城市健康发展提供了制度保证。按照组团式带状城市发展布局，制定了新老区连接带发展规划，并着力组织实施。完成城镇基础设施投资2.2亿元，较上年增长65.4%，城市功能进一步完善。城镇化率达到41.2%。新区续建、新开工道路12.1公里，主框架拉大到38平方公里，新增、改造绿地36万平方米，绿化率达到38.2%。北部工业区建设步伐加快。淇河新区段综合开发已经启动。市文化中心建成并向社会开放,新区文化功能得到较大增强。游泳馆基本具备开馆条件。王升屯村整体搬迁建设顺利推进。淇滨污水处理厂试运行。推进道路、污水处理、管网、绿化等基础设施建设，山城区、鹤山区环境和面貌得到进一步改观。强化城市管理综合执法，积极推进环卫、绿化等管理体制改革。新老区物业管理覆盖面分别达到91%和40.5%。两县县城努力控制零星住宅,启动住宅小区开发,进一步提高了城镇管理水平。21个村庄被确定为省康居建设示范村试点。积极争取建设用地指标，做好土地储备和治理整理工作,努力提高土地利用率和容积率。国有土地出让实现净收益5452万元，支持了城市建设和发展。

　　社会事业全面发展。加大对社会事业的投入，科技、教育、文化、卫生、体育事业的财政支出达到3.3亿元。列入国家、省科技发展计划项目31项，实施率100%。食品安全综合示范区项目、国家粮食丰产科技工程进展顺利。人才培养、引进工作取得新进展。基础教育稳步发展，新区高中、鹤壁外国语中学建成并开始招生，普通高中招生人数增长20%。被列入全国农村中小学远程教育工程试点市，全市668所农村学校参与试点。改造农村中小学D级危房2.95万平方米。职业教育、高等教育发展步伐加快，职业技术学院全日制大专在校生突破3000人。新世纪广场被评为全国特色文化广场。《七品知县卖红薯》荣获中国电影政府最高奖“华表奖”。浚县传统庙会文化列入国家民族民间文化保护工程试点。市120紧急救援指挥中心和传染病医院门诊楼、病房楼主体工程以及两县传染病区和2所中心乡卫生院建设项目完工。有效预防了高致病性禽流感，确保了我市境内无疫情。成功举办了市八运会。人口和计划生育工作水平稳步提高，人口自然增长率4.12‰。深入开展双拥工作，军政军民团结的大好局面进一步巩固。民族团结和进步事业继续发展。

　　精神文明和民主法制建设继续加强。深入开展“诚信在鹤壁，道德伴我行”活动和“双创”工作，被评为省“双创”工作先进市。不断加强未成年人思想道德教育，严厉打击黑网吧，严格规范网吧经营，净化未成年人成长环境。“扫黄打非”工作获得全国先进单位称号。广泛开展普法教育，公民的法制意识进一步增强，各级各部门依法行政能力得到提高。严格执行人大及其常委会通过的决定和决议，坚持向人大报告工作、向政协通报情况，认真办理人大代表建议35件、政协提案218件。积极推进政务公开，密切与工会、共青团、妇联等人民团体的联系，充分发挥政府网站、电视电台等媒体与广大市民的沟通作用，强化社会对政府工作的监督。下力气抓好信访工作，保持了在全省的先进位次。认真抓好社会治安综合治理，突出抓好命案侦破、打击“双抢”和入室盗窃、打击黑恶势力等工作，全市刑事和治安案件明显下降，命案积案侦破率、现行命案侦破率均居全省第一，人民群众对社会治安满意和基本满意率达到92.4%。坚持不懈抓好安全生产监管队伍和制度建设，加强道路交通安全管理，各类事故死亡人数下降11.3%，被评为全省安全生产先进市。

　　人民生活水平进一步提高。城镇居民人均可支配收入增长13.7%,达到6793元，在全省排名前移1个位次。农民人均纯收入增长18%，突破3000元，达到3014元，超过了全国平均水平。高度重视、认真解决关系群众切身利益的问题，年初向社会公开承诺的实事全部完成。“两个确保”工作得到巩固。养老保险扩面、养老保险费征收分别完成年目标的223%和102.4%。全市按时足额发放国有企业下岗职工基本生活费和企业离退休人员养老金1.43亿元。为57141名城市“低保”对象发放低保金4138.7万元，实现了动态管理下的应保尽保。浚县77%的农民参加农村新型合作医疗，累计为13万农民补助医疗费772万元。坚持集中救助与经常性救助相结合，组织3万余名干部、筹措900多万元资金物资对1万多户贫困家庭进行帮扶，建立了帮扶救助动态管理长效机制，初步构建了新型城乡社会救助体系。成立了市慈善总会，组建了慈善志愿者队伍，努力拓展慈善工作的新思路、新办法。大力开展劳务输出工作，全年劳务输出15.2万人，较年初增长154.8%，其中农民外出务工14.7万人，占农村剩余劳动力的69.5%，增加农民收入4.8亿元。把就业培训特别是技能培训作为促进就业再就业的重点工作来抓，全年免费开展就业再就业培训11448人，完成省目标的184.6%，是上年的5.25倍，经培训实现就业再就业7282人；城镇新增就业人员26530人，完成省目标的176.9%，居全省第一；下岗失业人员再就业14553人，完成省目标的207.9%，也居全省先进位次。粮食直补和农业税减免政策全面落实到位，1789万元补贴资金全部兑现，全市农民人均减负增收40.7元。深入开展服务“三农”集中活动，组织了大规模、长时间、多形式的科技下乡，培训农民50万人次，受到广大农民的普遍欢迎。解决农村饮水困难2.2万人。被省里确定为解决卫河沿岸群众饮水安全试点，一期工程启动。建设领域工程款和农民工工资拖欠的清理工作取得明显进展。投入381万元重建和修缮因灾倒房危房1429间，1715名受灾群众得到妥善安置。食品药品放心工程取得积极成效。关闭和停产整顿了一批污染严重的企业，强化了对淇河的保护管理，全市出境河流水质达到规定标准，市区环境空气质量二级标准以上天数的比例达到81.4%，饮用水源地水质达标率保持100%。

　　经济发展环境明显优化。以营造更高层次的发展环境为目标，扎实推进学习型、法治型、服务型政府建设。大力倡导和推动干部队伍的业务学习培训，形成了浓厚的学习氛围。以行政服务和办公自动化为重点，加快全市电子政务建设，电子政务开始试运行。在全市政府部门推进优化内部工作程序、提高工作效率、建立责任制并确保责任落实到人等工作，实行重点工作台帐制，从制度上解决政府部门工作棚架、低效运转、督查落实不力等问题。进一步完善市行政服务中心特别是窗口建设，提高了工作效率和服务水平。对招商引资重点项目实行了一系列切实有效的制度和办法，协调解决项目从落地、建设到经营过程中的各种问题。创造性地优化精简建设工程领域审批程序，审批时间由6个月减少到1个月以内。在全省率先试行建设领域行政事业性收费打捆收费。开展全方位效能监察，严肃查处破坏经济发展环境及效能方面的案件，共受理效能投诉案件115起，立案、查结48起，对9个领导班子、46名个人实施了效能责任追究。认真落实党风廉政建设责任制和领导干部廉洁自律各项规定，加大查办案件和纠风工作力度，廉政建设和反腐败斗争取得新成效。各级各部门工作质量、工作效率明显提高，为经济社会快速健康发展，特别是招商引资、大项目建设等工作取得突破提供了有力保证。

　　各位代表，2004年我市经济社会发展取得了令人鼓舞的好成绩，呈现诸多值得全市人民自豪的亮点。政府各部门有42项重点工作受到省以上表彰奖励。全市生产总值、人均生产总值、城镇固定资产投资、引进市外直接投资、农民人均纯收入5项指标实现了历史性突破，特别是完成了全面建设小康社会第一步战略目标,实现了“两个提前”的第一个提前。这些成绩的取得来之不易，是省委、省政府和市委正确领导的结果，是市人大、市政协积极支持的结果，更是全市广大干部群众克服重重困难、奋力拼搏的结果。广大干部志存高远、争创一流的坚定信念，解放思想、不断创新的开拓精神，知难而进、干事创业的顽强意志，已经成为鹤壁巨大的精神财富和加快发展的不竭动力。在此，我代表市人民政府，向工作在全市各条战线的广大工人、农民、知识分子、干部、人民解放军指战员、武警官兵、公安民警及社会各界人士致以崇高的敬意！向关心、支持我市现代化建设的朋友们表示衷心的感谢！

　　在取得上述成绩的同时，我们也清楚地看到，我市经济社会发展中还存在一些困难和问题，工作中还有不少需要加强和改进的地方。财政一般预算收入、规模以上工业企业增加值和综合经济效益指数的增速虽然取得我市多年以来的最好成绩，但仍低于全省平均水平；第三产业发展也较为滞后；支柱财源少、财政资金紧张、中小企业融资难状况尚未从根本上改观；城镇居民人均可支配收入和消费支出在全省的位次还比较靠后；改革发展稳定的任务仍然较重；少数部门和工作人员服务意识、工作质量和办事效率需要进一步提高，发展环境还有待提升到更高层次。我们将研究积极有效的措施，在今后工作中努力加以解决。

　　二、2005年工作总体要求和主要预期目标

　　2005年是我市全面完成“十五”计划、衔接“十一五”发展的重要一年，也是实现“两个提前”、确保“两个率先”的重要一年。我们要正确把握形势，积极应对发展中的困难和挑战，抢抓机遇,乘势而上，以更大的力度、更快的节奏、更高的效率、更好的服务，实现全市经济社会发展的新跨越。

　　今年政府工作的总体要求是：以邓小平理论和“三个代表”重要思想为指导，认真贯彻党的十六大、十六届三中、四中全会和省委七届八次全会精神，坚持以科学发展观统领经济社会发展全局，围绕实现“两个提前”、确保“两个率先”奋斗目标，继续强力推进招商引资、大项目建设，切实抓好县域经济发展、非公有制经济发展、城镇化、农民增收、服务型政府建设、各项改革等重点工作，大力发展循环经济，发展各项社会事业，改善城乡居民生活，努力构建和谐社会，促进经济社会全面协调可持续发展。

　　经济社会发展主要预期目标：全市生产总值努力实现增长16%以上；全社会固定资产投资完成70亿元，力争80亿元，其中城镇固定资产投资完成60亿元，力争70亿元；社会消费品零售总额增长14%；招商引资到位市外直接投资60亿元，力争80亿元，实际利用外商直接投资2000万美元，力争3000万美元；财政一般预算收入增长16 %以上；城镇居民人均可支配收入增长8%，农民人均纯收入增长6%；人口自然增长率控制在5‰以内；城镇新增就业3万人，下岗失业人员再就业8000人，城镇登记失业率低于4.5%。

　　三、加压驱动，拼搏争先，

　　努力实现经济发展的新跨越

　　(一)继续把招商引资放在重中之重的位置，加快开放型经济发展步伐

　　围绕建设“三大基地”和发展循环经济，抓好新项目的引进，抓好项目的落地与建设，突出抓好工业大项目，突出抓好技术含量高和对财政、就业贡献大的项目，力求招商引资数量的新突破、质量的新提高。

　　进一步增强各级各部门的紧迫感、责任感和使命感。不断总结招商引资经验，创新招商引资方式方法。坚持和完善市领导联系招商引资重点项目、全程代办、跟踪服务和联络员等制度，建立落地项目服务单位责任制，把责任落实到相关部门的分管领导、具体科室和有关人员，提高项目落地率，加快资金到位和工程建设进度。进一步树立全市招商引资工作一盘棋思想，加强相互学习、沟通与合作，继续搞好招商引资信息平台建设，真正实现全市招商引资资源共享。切实做好考核认定工作，及时兑现奖惩，充分调动各方面的积极性、创造性。

　　进一步明确主攻区域和方向。巩固长三角、珠三角、环渤海湾经济圈等招商基地；积极寻求与沿海地区开发区、工业园区以及企业商会、政府经济部门的沟通协作；加强与省内外有扩张计划、寻求发展空间的大企业的联系、交流与合作。加强境外招商工作，巩固和打开泰国、新加坡、美国、欧盟及香港、台湾等国家和地区的招商引资局面。引导支持各县区、市政府有关部门发挥各自优势，选准主攻地区和产业，扩大招商引资成果。各县区至少要有2个超亿元的工业项目落地，市直招商引资主要部门至少要有1个超亿元的工业项目落地。高度重视科技含量高、成长性强、见效快、就业容量大的中小项目的引进,通过小企业集群见规模、见效益。进一步加大旅游、教育、外向型市场等领域招商引资工作推进力度，促进第三产业发展。推动鹤壁-辉县-山西高平高速公路项目规划等前期工作,争取列入全省规划并早日实施。

　　进一步提高招商引资的层次和水平。紧紧围绕国家、省宏观政策和产业导向，把握国际国内产业发展转移规律和趋势，从中捕捉信息，寻找机遇，进一步做好承接境外和沿海地区产业转移的前期策划等基础工作，有的放矢外出招商，实现从一般化招商向个性化招商转变。把对落地企业的服务放在与引进项目同等重要的位置，进一步改善企业发展环境，吸引更多的后续投资和关联配套企业。对全市产业、资源、现有企业等情况进一步摸底梳理，发掘招商引资资源，培育招商引资载体。充分利用电力资源优势，吸引有较高技术含量的高载能产业和产品来我市发展。把鹤煤(集团)公司的招商引资摆上重要日程，鼓励和引导其把地面企业改革发展与招商引资紧密结合。调动企业作为招商引资主体的积极性、主动性，实现以政府直接招商为主向政府招商和以商招商并重的转变。把引进项目、资金与引进人才、智力更好地结合起来，提高利用外资的能力和水平，实现招商引资从以数量为主向数量与质量并重的转变。

　　(二)加快新型工业化步伐，着力转变经济增长方式

　　坚持建设“三大基地”的发展方向和目标，加快产业结构和布局调整，使资源型工业基地更具规模，先进制造业基地建设取得更快进展，食品加工业基地优势和特色更加明显。规模以上工业企业增加值增长22%；综合经济效益指数达到135%，接近全省平均水平。

　　继续把大项目尤其是工业大项目建设作为重要载体和抓手，千方百计加以推进，保持投资的高质量、高强度。在抓好现有投资亿元以上工业项目的同时，争取通过招商引资再上一批亿元以上项目，使投资亿元以上工业项目达到30个以上。电厂二期工程两台机组分别于5月、8月发电,加快推进电厂三期工程。综合利用热电项目争取完成土建工程和部分设备安装。水泥厂连续扩建三期工程建成投产。加快鹤煤(集团)公司四矿、九矿改扩建步伐，开工建设彰武-伦掌煤矿。整合全市有关资源，大力推进甲醇煤化工项目，力争尽快开工建设。做好长治-鹤壁输煤管线前期工作。

　　积极推进产业聚集和优化升级工作。强化工业集中布局观念，进一步搞好两县三区和开发区产业布局特别是优势产业发展布局规划。努力吸引同类企业、上下游及横向关联企业和产品向工业区集中，符合环保要求的大项目尽可能向新区及其周边集中，促进形成特色产业集群。通过2-3年的努力，在全市培育3-5个具有较大规模和竞争优势的特色产业。加快工业集中区基础设施建设，实行封闭管理，努力营造优良的发展环境。鼓励和支持鹤煤(集团)公司建设地面企业工业集中区。坚持扶优扶强，促进30户市重点工业企业发挥优势，尽快提高综合竞争力。加快金属镁、水资源、农产品、精细化工等优势资源的开发利用，在扩大规模、延伸链条、优化升级上力求突破，形成产业优势。积极引导和大力支持现有企业特别是传统优势企业运用高新技术和先进适用技术，加快产品、设备、工艺等改造升级，开发精深加工产品、终端产品和高端产品，打造省级、国家级名牌产品。进一步加强工业经济运行的预测、协调与服务。

　　抓住我市被确定为省循环型城市试点市的机遇，积极探索、扎实推进有关工作，争取列入国家循环型城市试点。引导全社会尤其是企业树立和强化节约能源、节约资源、保护环境的社会责任意识，努力建设节约型社会。加快构建和延伸煤炭、金属镁、水泥、畜牧养殖加工、秸秆综合利用等产业循环链，加快煤炭资源整合，积极推进鹤煤(集团)公司矿井水综合利用、维恩克公司环保节能硅热还原冶炼、同力水泥余热发电、秸秆发电等项目，在鹤煤(集团)公司进一步推广、建设煤层气发电项目。大力推广清洁生产，从源头上控制工业污染，实现所有工业企业主要污染物达标排放，做好农业面源污染防治，坚决防止新上不符合环保要求的项目，为新项目建设腾出更多的环境容量。积极稳妥做好关停年产10万吨以下立窑水泥生产线和年产1000吨以下金属镁冶炼企业工作。建设市环境监控中心。山城污水处理厂争取通过国家验收。淇滨污水处理厂加快配套工程建设。淇县污水处理厂试运行。浚县污水处理厂开工建设。市垃圾处理厂争取开工建设。

　　加快服务业发展。用先进理念和现代技术提升传统服务业。大力发展现代物流、新型商业业态、信息、金融、中介等现代服务业，积极培育新的消费热点。设立旅游发展专项资金，加强“一河(淇河)、两城(浚县国家历史文化名城、淇县朝歌古城)、三山(大伾山、云梦山、五岩山)”宣传推介和建设，更好地发挥中国优秀旅游城市品牌效应，使旅游业实现大的发展。在强化淇河保护的同时，把淇河综合开发与农业结构调整、发展休闲度假旅游等结合起来，采取有效措施使开发工作初见成效。督促县区在景区建设上加大旅游招商引资力度，确保重点景区景点建设取得明显进展。积极稳妥推进移民工作，建成盘石头水库，加快水库生态保护和旅游综合开发，实现水库工作从工程建设向工程管理及综合开发的转变。

　　(三)扎实做好“三农”工作，加快县域经济发展

　　切实把“三农”工作放在重要位置，认真贯彻中央一号文件和省加大对“三农”支持的政策，努力争取项目和资金支持，加快农业和农村基础设施、优势工业项目及城镇化、城乡市场体系等建设，提高农业综合生产能力，实现农业增效、农民增收。切实做好免征农业税、对种粮农民直接补贴以及增加良种补贴、农机具购置补贴等工作。交通基础设施建设完成投资2.3亿元。大海线大河涧至京珠高速公路段等3条干线竣工通车。做好农网三期县城电网改造工作。继续抓好食品安全综合示范区建设、农业综合开发和扶贫开发。组织实施国家粮食丰产科技工程、国家优质粮食产业工程农机装备推进项目，提高农业的科技含量和机械化水平。豫北粮食仓储物流交易中心二期工程投入使用。完成造林绿化10万亩，新建、完善平原林网20万亩。发展节水灌溉4万亩，治理水土流失43平方公里。

　　加快推进龙头带动战略，进一步提升农业产业化水平。继续扶持农业产业化龙头企业做大做强，扩大农产品绿色、安全、无害化、标准化生产，提高农产品加工转化能力，带动食品加工业基地建设和农业结构调整，使畜牧业、粮食、蔬菜及瓜果生产加工结构进一步优化，优势进一步显现。畜牧业产值达到35亿元，加工产值达到40亿元。力争普及优质小麦。完善服务“三农”长效机制，实行农业技术服务员和农村信息传递员制度，组织技术人员进村入户，培训农民50万人次以上。积极发展农民专业合作组织，加强农民经纪人队伍建设，提高农业、农村和农民的发展能力。

　　坚持市、县经济一体化发展，大力发展县域经济。认真贯彻省进一步扩大县级管理权限等政策规定，支持引导浚县、淇县进一步转变思想观念，创新发展思路，抓住机遇，加快富民强县步伐。督促两县坚持工业兴县主战略，加大招商引资力度，引导加工制造业向工业集中区聚集，着力培育优势特色产业，壮大县级财力，提升综合经济实力。与2003年相比，今年两县综合经济实力在全省排名各前移至少两个位次，一般预算收入增速争取超过全省各县的平均水平，淇县力争进入全省县域经济发展先进行列，浚县在加快农业县向工业强县转变上见到明显成效。

　　(四)坚持市、县、镇协调发展，加快城镇化进程

　　坚持以新区建设为龙头，市、县、重点镇三头并举，大力培育产业支撑，完善城市功能，提高管理水平。

　　以招商引资为主要手段，以工业发展为重点，积极发展休闲度假、社区服务、教育培训、文化、体育、旅游、外向型市场、现代物流等第三产业，强化产业支撑，增强城市功能。抓住国家和省拓宽安新高速公路、建设郑州至北京铁路客运专线和南水北调中线工程的机遇，筹划有关配套和服务项目，拉动我市相关产业发展。

　　加强城市规划、建设和管理，积极开展“双创”工作，创建“中国人居环境范例奖”。建设新区城市道路、采煤沉陷治理、污水处理等项目，总投资13.2亿元，年内计划完成投资4亿元。一是进一步完善新区规划和总体设计，完善功能，丰富内涵，提高品位。做好土地利用工作，实现规模扩张和集约式发展的有机结合。北部以工业区发展为重点，加快基础设施建设，完善服务功能，尽快成规模、见成效；西部以淇河新区段开发为重点，加快推动湘江路、滨河东路、沿河步行道建设和淇滨乐园开发等工作；中部建成区以完善教育、文化等重要城市功能和建设商务中心区为重点，进一步提升城市品位。以新区及周边产业发展和快速通道建成通车为契机，加快新区人气聚集，促进新区人流物流的增加和房地产市场景气的提升。根据市场需求的变化，适时推出具有更高品位和档次的住宅小区以及商用写字楼建设。高标准高品位做好绿化美化工作，实施生态型、多样性绿化，重点部位打造精品，新增、改造绿地面积29万平方米。进一步完善城市道路框架。完成王升屯村改造搬迁，推进大赉店等城中村的改造建设。做好火车站整治改造工作。改革供热体制和模式，完善供热管理办法，提高供热质量。规划建设新区北部工业区、金山工业区、钜桥工业区供热管网。二是科学确定山城区、鹤山区的发展定位，立足长远谋划和推动其建设与发展。在产业发展上，以建设资源型工业基地及关联配套产业为主体，兼顾传统优势产业的改造升级和服务业的档次提升；在空间规划布局和发展模式上，以工业布局为主体，控制以适度的人口规模和城区规模。在强化产业扩张的同时，城区建设以改善基础设施和居住环境为重点，走内涵式发展的路子。为此，要认真做好规划工作，加强规划执法管理，制止无序开发。结合采煤沉陷治理、关闭污染企业、启动燃煤小锅炉治理等工作，加强城区环境整治和城区周边生态建设，并通过进一步做好城区绿化、美化工作和道路、管网等基础设施建设，努力改善城区环境，提高居民生活居住质量。三是创新城市管理方式，加强城市综合执法和社区建设，加大对乱停乱放、交通违章、超限遗撒等治理力度。搞好小区和背街小巷管理，规范、完善物业管理。四是引导、督促两县加快做好县城规划，并努力加强规划管理，结合县城改造和产业发展认真做好土地利用工作，积极推进县城住宅小区开发，坚决制止零星住宅建设。搞好重点小城镇的规划、策划，着力培育产业支撑，积极探索行之有效的小城镇建设模式，形成各自的特色和优势。

　　严格土地保护和管理，规范土地出让方式，提高土地集约利用水平。科学制订全市工业发展和城市建设用地规划。加强新区土地的规划、整理、管理与开发。抓好县城和重点小城镇土地的整理和利用，加大日常监督力度，严肃查处土地违法事件。搞好淇河两岸、快速通道和大白线两侧土地的规划、控制和开发。科学确定建设项目用地规模，鼓励新上项目利用现有建设用地，积极推广多层标准厂房。

　　(五)深化各项改革，不断推进体制和机制创新

　　全面完成国有企业改革。继续把企业改革与招商引资、发展非公有制经济等紧密结合,努力吸引更多有实力的投资者参与改革。对已改制企业继续搞好引导和服务,促进企业真正转变观念，引入新机制、新技术、新产品，提高市场竞争能力。对尚未改制的企业实行一企一策，把改制工作做细做实做好。鼓励、引导和服务好鹤煤(集团)公司地面企业改革。

　　加快投融资体制改革。明确政府投资范围，落实企业投资决策自主权，鼓励、引导和拓宽社会投资领域。规范政府投资行为，扎实推进政府投资项目代建制，科学规范程序，严格项目概算和工程管理。协助和推动鹤壁煤电股份有限公司做好上市工作，鹤煤(集团)公司尽早发行10亿元企业债券，维恩克、大用等公司开展上市前期工作。做好农村信用社改革后期工作。加快城市信用社增资扩股、清收不良贷款等工作，努力向商业银行方向发展。进一步发挥市担保中心的担保融资作用，努力吸引市外资金设立投资担保公司和创业投资公司，支持我市中小企业发展。进一步促进银企合作，建立银企互信互利关系，更大限度地发挥金融机构对企业的支持作用。支持金融机构加快改革,加强内部管理，发展中间业务，增强服务能力,加快发展步伐。

　　深化财政体制改革。全面推行乡镇财政管理方式改革。加强水资源费、污水处理费、矿产资源费等征收工作。整合市级财政专项资金，集中财力支持重点项目和重点工作，提高资金使用效益。坚持依法加强税收征管与积极培植财源相结合,确保财政收入稳定增长。进一步严格和规范各级政府和部门债务资金使用，建立偿债准备金并纳入预算,同时通过多种方式筹措资金，逐步减少市直部门和开发区的债务负担。加大争取资金力度,力求更大的突破。县区政府也要根据自身实际情况，重视和做好政府债务管理工作。

　　进一步深化事业单位人事制度改革和乡镇机构改革。推进市政公用事业经营管理体制改革。整合医疗卫生资源，深化医疗卫生体制改革。平稳推进和基本完成国有粮食购销企业改革。加快文化体制改革步伐。抓好干线公路养护、交通规费征收、道路运输场站管理体制改革。

　　(六)坚持“放心、放开、放手”的方针，促进非公有制经济更快更好地发展

　　认真贯彻国家、省优惠政策，进一步完善支持非公有制经济发展的政策措施，努力创造有利于非公有制经济发展的环境。创新方式，在信息、人才、技术、员工培训等方面搞好对非公有制企业的服务。鼓励和引导金融机构加大对有信誉、有发展潜力的中小企业的支持力度。多途径增强市担保中心的担保能力，努力帮助中小企业解决融资难问题。引导和督促非公有制企业依法经营，诚实守信，更好地履行社会责任，谋求长远发展。鼓励非公有制企业树立现代企业管理理念，创新管理模式，提高管理水平。支持有条件的企业加大科技投入，增强研发能力，不断提高产品档次和产业层次，尽快做强做大。力争非公有制经济在数量、质量上实现新突破，占全市生产总值的比重达到49%以上。

　　四、统筹经济社会发展，努力构建和谐社会

　　切实做好就业再就业和社会保障工作。注重发展劳动密集型企业，创造更多的就业岗位，努力在发展中解决就业问题。把下岗失业人员、新增劳动力、需要就业的失地农民统一纳入就业再就业工作范畴，全面摸清就业需求底数，实施分类管理。突出抓好就业再就业技能培训，发挥政府在培训中的主导作用，整合培训力量，加大培训力度，增强培训效果，提高就业质量。改善农民工进城就业环境，保障其合法权益。继续做好劳务输出，鼓励引导外出务工人员回乡创业。力争用2年时间，使我市劳动就业形势实现根本好转，带动城乡居民收入和消费水平明显提高。继续做好社会保险扩面、“两个确保”和城市“低保”工作。实现企业职工基本养老保险市级统筹。做好国有企业下岗职工全部出再就业服务中心工作，实现国有企业下岗职工基本生活保障向失业保险并轨。完善城镇职工基本医疗保险制度。进一步做好浚县新型农村合作医疗试点工作。提高社区在就业和社会保障方面的服务能力，企业退休人员全面实行社会化管理。建立和完善新型城乡特困群众救助体系，努力使特困群众得到及时帮扶救助。完善慈善捐助管理服务机构，扩大志愿者队伍，开展经常性捐赠，积极发展慈善事业。

　　高度重视信访稳定和安全生产工作。认真贯彻国家新颁布的《信访条例》，落实各项信访制度和措施，积极预防和妥善处理群体性事件。抓好基层基础信访工作，充分发挥基层组织第一道防线作用。探索建立下访活动长效机制。加强法律宣传，引导群众通过法律程序解决涉及利益诉求和涉法信访问题。继续保持信访工作在全省先进位次，争创全国信访工作先进单位。进一步充实基层警力，加强社会治安综合治理和防控体系建设，始终保持对刑事犯罪的高压态势，确保刑事、治安案件继续明显下降，保持良好的社会治安状况，争创省社会治安综合治理先进市。坚持把安全工作放在十分重要的位置，进一步发挥安全生产网络体系的作用，落实责任制，抓重点领域，抓薄弱环节，把每一项工作、每一个环节抓细抓实，坚决防止重特大安全事故的发生，实现事故起数和死亡人数“双降”，保持安全生产工作在全省的先进位次。

　　全面发展各项社会事业。完善、落实人才政策，进一步做好人才特别是企业家的培训、引进工作。充分发挥人才市场作用，为人才交流和引进提供便利场所和优质服务。树立科学的教学观，从教育行政部门、学校、老师、学生和家长五个环节入手，进一步抓好教学管理，重视学生素质教育，提高教学质量。整合教育资源，积极探索通过公办民助、民办公助等方式发展义务教育和高中阶段教育。围绕市场需求，突出专业特色，积极发展职业教育和高等教育。职业技术学院积极开展对外合作，多渠道筹措资金，为争取通过国家教育评估验收创造条件。新区工贸学校主体工程完工并具备招生条件。继续抓好中小学乱收费治理工作，严格执行“一费制”。加快文化事业和文化产业发展。加强突发公共卫生事件应急处置特别是医疗救治体系建设。优化公共卫生资源配置，加大对农村卫生事业的投入，完善公共卫生体系。加强重大传染病预防控制。继续抓好食品药品放心工程。广泛开展全民健身活动。努力促进体育产业的发展。进一步提高广播电视“村村通”水平。做好第一次全国经济普查。加强人口和计划生育工作，稳定低生育水平，提高出生人口素质。深入开展国防教育，进一步加强民兵预备役建设，落实军人抚恤优待政策，做好军队转业干部、退役士兵安置和拥军优属工作，增强军政军民团结。

　　加强精神文明和民主法制建设。全面落实公民道德建设实施纲要，深入开展社会公德、职业道德和家庭美德教育，努力在全社会形成良好道德风尚。继续加强未成年人思想道德建设。加强诚信教育，倡导诚实守信，推进全社会信用体系建设。积极开展法律宣传、科技、文化、卫生进社区和“四下乡”活动，继续搞好社区文化、村镇文化、企业文化、校园文化建设，进一步丰富群众文化生活。坚持依法治市。加强法制宣传教育，提高城乡居民法律素质和依法行事的意识。严格执行人大及其常委会通过的决定和决议，接受其法律监督和工作监督。支持人大代表依法行使职权。接受人民政协的民主监督。认真办理人大代表建议和政协提案。广泛听取民主党派的意见，充分发挥工会、共青团、妇联等人民团体及工商联、商会等社团组织的桥梁纽带作用。提高政府工作的公众参与度，更充分地发挥专家学者的作用，努力提高决策质量。继续做好民族宗教工作。依法保护妇女儿童、残疾人和未成年人的合法权益。巩固和扩大基层民主，完善村民自治、社区民主管理和企业民主管理制度。做好第五届村民委员会换届选举工作。

　　集中政府财力，做好与群众生活密切相关的10件实事：

　　1、投资4600万元，解决卫河沿岸1.5万人饮水安全问题，修建县乡道路111公里，改善农村生活生产条件。

　　2、投资4700万元，改造农村中小学D级危房3.38万平方米，完成农村中小学远程教育工程试点建设，整合山城区、鹤山区中小学资源，改善学校教学条件。

　　3、投资4000万元，建设嵩山路中学、鹿鸣小学、福田小学，满足新区不断增长的就学需求。

　　4、投资1000万元，实现疾病预防控制中心交付使用，启动肿瘤医院门诊楼和妇幼保健院门诊楼建设，完成3所中心乡卫生院、4所乡镇卫生院改造建设,使我市公共卫生体系进一步完善。

　　5、投入2300万元，开展6500名下岗失业人员和6000名农民工技能培训，建设、完善新老区劳动力市场；建设社会保险信息系统项目；为6000余名困难企业退休职工提供大病救助和大病保险。

　　6、投资800万元，建设儿童福利院、老年康复中心和救助管理站，提升我市社会福利事业水平。

　　7、投资2.4亿元，启动采煤沉陷治理工程，开工建设2个住宅小区，完成受损医院和学校择地重建及2000农村受损户补偿工作。

　　8、投资4200万元，改造、建设新区部分供热管网，完成二支渠改造、护城河开挖，初步形成淇河水引入城区的循环水系；实施老区道路、管网等基础设施建设和亮化、美化工程，改善市民居住条件和生活环境。

　　9、投资800万元，开工建设新区体育中心(体校)部分场馆，做好山城区体育场新址规划和建设工作。

　　10、投入500万元，建立完善市县乡三级便民行政服务网络，整合行政服务监督热线电话，健全政府服务体系，提高服务质量。

　　五、切实转变工作作风，进一步提高政府工作水平

　　我市经济转型速度很快，工作环境变化很快，工作要求提高很快。新时期、新形势、新任务，要求我们进一步加强政府自身建设，使之成为运转高效的政府，依法行政的政府，真正为人民负责任的政府。各级政府及全体工作人员特别是各级领导干部，要进一步解放思想，更新观念，开拓进取，把各项工作做实做好；要进一步增强开放意识，积极走出去、请进来，拓展视野，增长见识，打破习惯思维，反思习惯做法，提高创新能力和管理水平；要进一步树立大局观念，切实摒弃部门之见和小团体利益，加强部门之间的协调配合，提高政府运转效率；要进一步强化服务意识，摒弃传统的、狭隘的权力观念，真正将管理寓于服务之中。要进一步加强干部的业务培训，提倡调查研究，营造努力学习、勤于思考、善于总结的工作氛围，使广大干部职工的潜力不断得到发掘，业务能力不断提高，使各项工作不断创新，干出特色，形成亮点。

　　全面推行市县乡三级政务公开，提高公开的质量和效果。加强市行政服务中心和第二行政服务中心窗口建设，加强山城分中心的建设和管理。继续优化部门内部工作流程，规范行政行为，提高工作效率，并以此为内容开展效能评价。规范建设领域行政事业性收费打捆收费制度。抓好电子政务运行，实现市、县区行政服务网上审批、督查和主要部门主要业务办公自动化。实行重点工作台帐制，保证对重点工作的有效督导和及时推进。立足当前，谋划长远，做好“十一五”规划编制工作。

　　牢固树立和落实科学的发展观和正确的政绩观，加强政风建设。坚持以人为本、执政为民，多做打基础、管长远、惠百姓的工作。教育引导广大干部职工围绕“实”字做文章，敬业奉献，不事张扬。勇于担当重任，勇于承担责任，不回避矛盾和工作难题。继续开展全方位效能监察，严肃查处破坏经济发展环境及效能方面的案件，为经济发展创造更加优良的环境。就政府部门对其下级部门和单位的管理、服务现状进行调查摸底，建章立制，规范管理，强化服务，在政府系统内部营造良好的工作环境。进一步树立市政府在工作部署上的权威，强化对市政府各部门、各县区的督导督查，做到有令则行、有禁必止，确保市政府工作部署和要求得到有效落实。加强经济监督，实施经济责任审计。牢记“两个务必”，坚持廉洁从政、从严治政，坚决反对、预防和惩治腐败。

　　各位代表，当前我市经济社会发展正处在一个重要的战略机遇期。中央、省加强和改善宏观调控，从政策、资金、重大项目布局等方面，进一步加大对中部崛起、“三农”等支持力度，有利于我市积极争取项目和资金，加快工业、农业、服务业以及能源、交通等基础产业发展。东部沿海地区产业、资金、技术和人才加快向中西部地区转移，有利于我市更好地发挥区位、资源、劳动力等优势，扩大招商引资效果。更为重要的是，经过全市人民的不懈努力，我市经济呈现不断加速发展的良好态势。全市生产总值从1995年到2002年7年时间增加50亿元，而2003年、2004年2年时间就增加51亿元。全社会固定资产投资从1999年到2002年4年间总和为77亿元，而2003年、2004年2年总和就达到80亿元。财政一般预算收入从1995年到2001年6年时间增长1.9亿元，而2001年到2004年3年时间增长2.1亿元。全市引进市外直接投资2002年是1.76亿元，2003年达到12.3亿元，2004年达到50.6亿元。这充分表明，我市经济总量和人均水平已经跃上一个新的战略起点，进入全新发展时期，展现在我们面前的是一个光辉灿烂的前景。我们完全有信心也有能力在新的一年里使各项工作再上一个新台阶，继续保持当前来之不易的十分良好的发展势头。

　　各位代表，面对当前经济社会发展的大好形势，我们不敢有丝毫自满；面对今年光荣而艰巨的目标任务，我们不敢有半点懈怠；展望我市经济社会发展的美好未来，我们充满信心。让我们以“三个代表”重要思想为指导，在省委、省政府和市委的正确领导下，锐意进取，扎实工作，为建设富裕、和谐、美丽的新鹤壁，为全市人民的福祉而努力奋斗！
